# How many genes might underlie QTLs for growth and wood quality traits in *Eucalyptus*?

**DOI:** 10.1186/1753-6561-5-S7-P37

**Published:** 2011-09-13

**Authors:** Carolina Sansaloni, César Petroli, Georgios Pappas, Orzenil Da Silva, Dario Grattapaglia

**Affiliations:** 1 EMBRAPA Genetic Resources and Biotechnology - EPqB Final W5 Norte 70770-917 Brazilia DF and Dep. Cell Biology, Universidade de Brazilia – UnB, Brazil; 2 EMBRAPA Genetic Resources and Biotechnology - EPqB Final W5 Norte 70770-917 Brazilia DF, Brazil; 3 EMBRAPA Genetic Resources and Biotechnology – Estação Parque Biológico, 70770-910, Brazilia, DF and Genomic Sciences Program - Universidade Católica de Brasília - Brazilia, Brazil

## Background

QTL mapping is an unbiased approach where the phenotype reveals the location of regulatory genes or genomic regions affecting the trait of interest. The development of transferable molecular markers and the increased use of multiple pedigrees for QTL mapping have allowed comparative analysis of QTLs across independent studies thus providing validation data. Such QTL positional information, together with the availability of annotated genome sequences, now promises to identify strong candidate genes for a number of traits [1]. As large pedigrees become available and higher resolution mapping with SNPs, DArT and genotype-by-sequencing technologies becomes routine in forest trees, QTL positional information could be an alternative to the current approaches that rely on tentative candidate genes for association genetics studies. Among the several traits for which QTLs have been mapped in forest trees those that display higher heritability, such as wood chemical composition, are more likely to involve candidate genes of stronger effect although recent association studies show that even such genes explain very small proportion of the variation [2]. In this study we used a high-resolution map with over 2,000 Diversity Arrays Technology (DArT) markers to carry out an initial assessment of the number of annotated gene models in the reference genome sequence of Eucalyptus that putatively co-locate with QTLs for growth and wood quality traits.

## Methods

A QTL mapping study was carried out with a clonally replicated segregating population of 171 F1 individuals derived from an *E. grandis* x *E. urophylla* cross. Individuals were genotyped with the Eucalyptus DArT microarray described earlier [3]. The DArT marker data were combined with 222 microsatellites and a linkage map for each parent was constructed using JoinMap 3.0 [4]. Six traits were measured: height growth (HG), circumference at breast height (CBH); wood specific gravity (WSG); cellulose pulp yield (%PULP); Total Lignin (TL); syringyl/guaiacyl ratio (S/G). QTL mapping was carried out using QTL Cartographer [5] on the two parental maps separately at 1 cM intervals. Empirical threshold significance levels for QTL detection were determined by 1,000 permutations considering a significance level of 5%. All the segregating DArT and microsatellite markers were mapped onto the 11 pseudo-molecules of the *Eucalyptus grandis* draft genome sequence covering 609 Mbp.

## Results

QTL analyses were carried out using framework genetic linkage maps with high likelihood support for order. The maternal map had 825 markers (684 DArTs + 141 SSR) and the paternal map 511 markers (410 DArTs + 101 SSR). A total of 16 QTLs in *E. grandis* and 14 in *E. urophylla* were detected influencing growth and wood quality traits. High and significant positive phenotypic correlations were found between CBH and HG, TL and S/G, and S/G and %PULP. In the maternal *E. grandis* map, five QTLs were identified for TL (Linkage groups (LG) 1, 3, 4, 5 and 8), two QTLs for %PULP (LG 4 and 5), three for S/G (LG 1, 5 and 8), WSG (LG 6, 8 and 10) and HG (LG 1, 2 and 6). In the *E. urophylla* paternal map we detected three QTLs for %PULP (LG 1, 4 and 9), three for HG (LG7, 8 and 10), three for TL (LG3, 4 and 8), two for CBH (LG7 and 10), two for S/G (LG 8 and 9), and one for WSG (LG 8). More than two QTLs were clustered on LG 4, 5 and 8 in *E. grandis* and on LG 4, 8 and 9 in *E. urophylla* suggesting interesting genomic regions to look for candidate genes to be tested in association mapping. Several of these QTLs were syntenic to QTLs found in other studies [6,7] providing some indirect support for their validity. The sequences of DArT and microsatellite markers bracketing QTLs were used to extract the gene models from the Eucalyptus reference genome. A total of 7,125 predicted gene models are found across all maternal QTLs, with an average of 445 genes per QTL. For the paternal QTLs 5,076 gene models exist, with an average of 362 genes per QTL.

## Conclusions

As in many other QTL mapping studies in Eucalyptus [6-8] we have identified several QTLs that control a modest proportion of the phenotypic variation for a number of economically important traits. In this first assessment of putative candidate genes co-locating with these QTLs we found thousands of annotated gene models, hundreds of which could be tentatively suggested as being involved in trait variation. Notwithstanding the low mapping resolution provided by the small progeny, this preliminary study shows that tens or hundreds of genes will likely be always found underlying QTLs for such complex traits. Testing and validation of such large numbers of genes will require a gigantic effort. Furthermore a large proportion of the phenotypic variation remains unexplained by the few QTLs mapped. It is therefore questionable from the applied stand point how much useful information this approach will effectively provide for the advancement of association genetics and, for that matter, of breeding practice.
